# Supplementary material for: Estimating total human deaths from rabies: A statistical framework leveraging reports of rabies transmission from deceased organ and tissue donors
Source: PLoS Negl Trop Dis. 2026 Sep 21;20(9):e0014731. doi: 10.1371/journal.pntd.0014731 (PMC13592719; doi:10.1371/journal.pntd.0014731)
Supplement: Supplementary file 6 [file pntd.0014731.s005.zip › Result_B/Cuba/Figure_S1_Cuba.pdf]

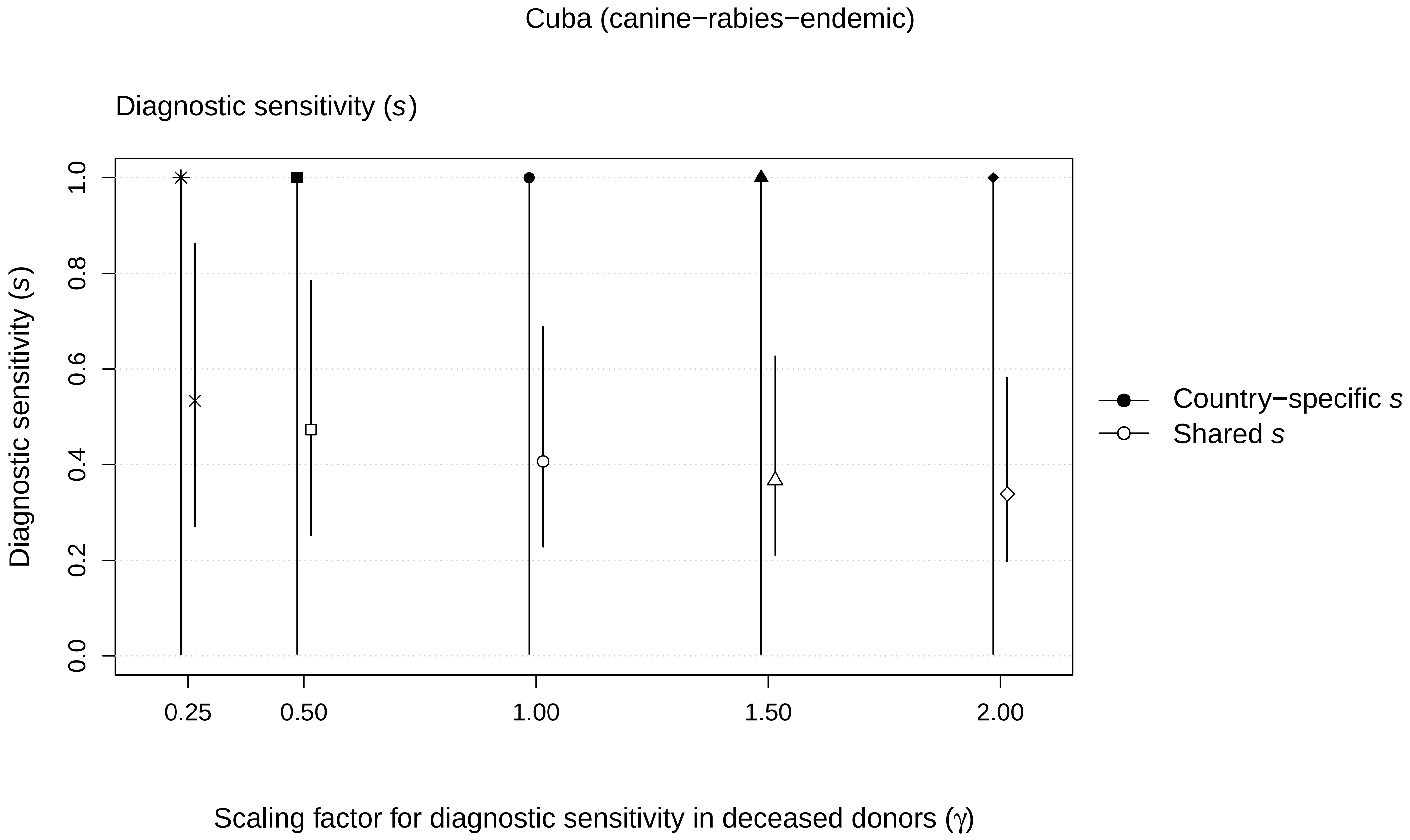

Cuba (canine−rabies−endemic)
Diagnostic sensitivity (*s*)
Diagnostic sensitivity (*s*)
0.0
0.2
0.4
0.6
0.8
1.0
0.25
0.50
1.00
1.50
2.00
Country−specific *s*
Shared *s*
Scaling factor for diagnostic sensitivity in deceased donors (γ)
